# Lung cancer and interstitial lung diseases: the lack of prognostic impact of lung cancer in IPF

**DOI:** 10.1007/s11739-021-02833-6

**Published:** 2021-09-15

**Authors:** Loredana Carobene, Donatella Spina, Maria Giulia Disanto, Claudio Micheletto, Maria Antonietta Mazzei, Piero Paladini, Claudia Ghiribelli, Elena Bargagli, Paola Rottoli

**Affiliations:** 1 Cardio-Thoracic Department, Respiratory Unit, Verona Integrated University Hospital, University of Verona, Square Aristide Stefani, 1, Verona, Italy; 2 Pathology Unit, Siena University Hospital, Siena, Italy; 3 Diagnostic Imaging Unit, Siena University Hospital, Siena, Italy; 4 Thoracic Surgery Unit, Siena University Hospital, Siena, Italy; 5 Respiratory Disease and Lung Transplant Unit, Siena University Hospital, Siena, Italy

**Keywords:** Lung cancer, Interstitial lung disease, Idiopathic pulmonary fibrosis, Survival

## Abstract

Lung Cancer (LC) is the first cause of death worldwide. Recently increased interest in interstitial lung diseases (ILD) has highlighted an association with lung cancer, offering interesting insights into the pathogenesis of the latter. Describe the association between lung cancer and ILD and evaluate the impact of LC on survival in these populations. We collected clinical, radiological, histologic data of 53 cases of advanced pulmonary fibrosis with lung cancer: 17 with UIP pattern (usual interstitial pneumonia, UIP/IPF-LC) and 36 with non-UIP pattern (ILD-LC). Adenocarcinoma was the most frequent histological subtype of lung cancer in all three groups and in UIP/IPF-LC developed in the lung periphery and in an advanced fibrosis context. Patients with DLCO% < 38% showed survival < 10 months, irrespective of group and development of carcinoma in UIP/IPF does not necessarily affect survival, unlike in SR-ILD. Our results confirm that the oncogenic mechanism is closely linked to fibrotic and inflammatory processes and that the development of carcinoma affects survival in SR-ILD but not in IPF.

## Introduction

Lung Cancer (LC) is the first cause of death worldwide and its association with smoking, occupational exposure and environmental pollution are known facts [1]. In recent years, increased interest in interstitial lung diseases has highlighted an association with lung cancer, offering interesting insights into the pathogenesis of the latter.

According to the literature, idiopathic pulmonary fibrosis (IPF) patients are at higher risk of LC than the general population, due to changes in lung architecture [2]. The incidence of lung cancer in IPF patients ranges from 4.4% to 48% [3], but there are little data on its incidence in patients with other types of interstitial lung disease (ILD) [3–8]. We know that in IPF patients, LC generally occurs in fibrotic areas of the lung, where normal lung architecture has been replaced by destructive fibrosis and honeycombing. We also know that squamous cell carcinoma is the most frequent histological type in these patients [9]. For other ILDs, little data are available [9].

We, therefore, collected and analyzed the features of a cohort of patients with IPF and other ILDs who developed or did not develop lung cancer. Our aim was to describe and seek insights into this association and to evaluate the impact of LC on survival in these populations.

## Materials and methods

### Study population

In this retrospective study, we evaluated 53 cases of advanced pulmonary fibrosis with lung cancer: 17 with UIP pattern (usual interstitial pneumonia, UIP/IPF-LC) and 36 with non-UIP pattern (ILD-LC). The latter group included 17 smoking-related ILD-LC cases (SR-ILD-LC) and 19 other ILD-LC cases (O-ILD-LC). The former (SR-ILD-LC) included respiratory bronchiolitis interstitial lung disease and combined pulmonary fibrosis and emphysema. The latter (O-ILD-LC) included chronic hypersensitivity pneumonitis, idiopathic and due to rheumatoid disease nonspecific interstitial pneumonia, pleuroparenchymal fibroelastosis, sarcoidosis, pneumoconiosis, lymphoid interstitial pneumonia and familial fibrosis. All patients gave informed consent to participation in the study that was approved by our Local Ethics Committee. All had a histological or cytological diagnosis of lung cancer, except five who only had a radiological diagnosis because invasive procedures were impossible due to their severe respiratory condition. Medical records of patients were collected in the Departments of Pulmonology, Thoracic Surgery and Pathology of the University Hospital of Siena. A database was built that included demographic data, family history of pulmonary fibrosis, smoking history, occupational/environmental exposure, lung function tests, tumor markers, comorbidities, and radiological patterns of interstitial lung involvement, cancer localization and histological features.

Lung function tests were performed according to ATS/ERS guidelines [10], recording forced vital capacity (FVC%) and diffusing capacity of the lung for carbon monoxide (DLCO%). Diagnosis of IPF was according to ATS/ERS guidelines [11]. High-resolution computed tomography (HRCT) of the chest was performed in all patients. Diagnosis of IPF and ILD were made in a multidisciplinary context, as required by recent recommendations [11]. Multidisciplinary discussion involved clinicians (pulmonologist, rheumatologist, and occupational physician), radiologist and pathologist. Patients with IPF were treated with nintedanib [12] or pirfenidone [13], according to drug inclusion/exclusion criteria.

Survival was evaluated and the vital status of patients was obtained by phone call or from medical records.

We also collected clinical data of 65 patients with ILD who did not develop lung cancer. We divided these patients as follows: UIP pattern (UIP/IPF, 38 cases), smoking-related ILD (SR-ILD, 6 cases), other ILD (O-ILD, 21 cases).

Demographic characteristics of patients who were not affected by lung cancer were superimposable to patients with ILD affected by lung cancer, for sex and age.

### Pathology

Lung fibrosis and cancer were evaluated in all available histological samples. Tumor histotypes and grades were defined according to WHO 2015 criteria on the basis of morphological and immunophenotypic characteristics [14]. Lung cancer stage was evaluated according to the TNM classification of lung cancer (8th edition) [15].

Cancer was classified as central if it developed around large bronchi, and peripheral if it was in the subpleural parenchyma. We evaluated the relationships between lung cancer and ILD. If it developed in areas where fibrotic tissue had subverted and replaced normal lung architecture, it was considered to be in a context of lung fibrosis. On the other hand, when it occurred in areas with preserved lung parenchyma, it was considered to be unrelated to fibrosis.

### Statistical analysis

Quantitative clinical characteristics were expressed as median and range. Significance was set at 95% (*p* = 0.05). The Kruskal–Wallis test was used to compare quantitative variables between groups. The Chi Square test was used to compare qualitative variables between groups. The Pearson coefficient was used to express correlations between quantitative variables. Survival was evaluated by the Kaplan–Meier test. Statistical analysis was conducted with IBM SPSS Statistics 20 software.

## Results

### Clinical findings in lung cancer patients

The population was composed of 17 UIP/IPF-LC (12 males, 5 females), 17 SR-ILD-LC (16 males, one female) and 19 O-ILD-LC cases (13 males, 6 females). No significant differences were found with regard to sex and smoking habits Table 1.

**Table 1:** Clinical findings of patients with lung cancer

Parameter	UIP/IPF-LC (Number or Range and Median)	SR-ILD-LC (Number or Range and Median)	O-ILD-LC (Number or Range and Median)	p UIP/IPF-LC vs SR-ILD-LC p UIP/IPF-LC vs O-ILD-LC p SR-ILD vs O-ILD-LC
*Sex*				0.087
Male	12	16	13	0.588
Women	5	1	6	0.052
*Smoke*				0.077
Yes	12	14	14	0.212
No	5	0	2	0.256
*Diagnosis*				
Histological	7	10	17	0.357
Cytological	8	4	2	0.008
Other	2	3	0	0.068
*Number of LC*				0.500
Single	12	11	11	0.330
Multiple	5	6	8	0.676
*Stage of LC*				(I + II vs III + IV)
I	4	7	13	0.720
II	2	0	2	0.008
III	5	6	2	0.020
IV	6	4	2	
Age at fibrosis diagnosis	599–982	633–967	507–918	0.984
Months (Years)	820 (68.3)	794 (66.16)	728 (60.66)	0.053
				0.026
Age at LC diagnosis	680–982	772–1063	544–918	0.922
Months (Years)	890 (74.16)	807 (67.25)	730 (60.83)	0.018
				0.040
FVC% at fibrosis diagnosis	49–117, 79	66–114, 81	66–104, 90	0.374
				0.484
				1.000
FVC% at LC diagnosis	37–112, 69	49–103, 71	55–124, 87	0.667
				0.121
				0.350
DLCO% at fibrosis diagnosis	15–68, 35	32–70, 45	34–69, 47	0.098
				0.080
				0.693
DLCO% at LC diagnosis	21–68, 31	10–61, 38	23–80, 43	0.462
				0.060
				0.219

Abbreviations *UIP/IPF-LC* pattern usual interstitial pneumonia with lung cancer, *SR-ILD-LC* smoking-related interstitial lung diseases with lung cancer, *O-ILD-LC* other interstitial lung diseases with lung cancer, *LC* lung cancer, *FVC* forced vital capacity, *DLCO* diffusing lung capacity for carbon monoxide

Lung cancer was diagnosed predominantly on the basis of cytological samples in the UIP/IPF-LC group and histological samples in the O-ILD-LC group (*p* = 0.008). The number of neoplastic lesions in the three groups was: one in 12 UIP/IPF-LC cases; one in 11 SR-ILD-LC cases; one in 11 O-ILD-LC cases; multiple in five UIP/IPF-LC cases; multiple in six SR-ILD-LC cases; multiple in eight O-ILD-LC cases. No significant differences were observed.

In the UIP/IPF-LC group, we recorded stage IV in six, stage III in five, stage II in two and stage I in four patients. In the SR-ILD-LC group, we recorded stage IV in four, stage III in six and stage I in seven patients. In the O-ILD-LC group, we recorded stage IV in two, stage III in two, stage II in two and stage I in 13 patients.

Early stages (I and II) were prevalent in O-ILD-LC, whereas more advanced stages (III, IV) prevailed in UIP/IPF-LC and in SR-ILD-LC cases (*p* = 0.008).

The median age at diagnosis of fibrosis and LC in the three groups was as follows: UIP/IPF-LC, 820 months (68.3 years) and 890 months (74.16 years); SR-ILD-LC, 794 months (66.16 years) and 807 months (67.25 years); O-ILD-LC, 728 months (60.66 years) and 730 months (60.83 years), respectively. Patients in the UIP/IPF and SR-ILD groups were older than those in the O-ILD group at diagnosis of fibrosis (p = 0.053) and at diagnosis of cancer (p = 0.018). The interval between the two diagnoses did not differ significantly between groups.

Patients with O-ILD-LC had better lung functional parameters (FVC% and DLCO%) than those with UIP/IPF-LC, but the difference was not significant. FVC% and DLCO%, normalized for time elapsed (ΔFVC and ΔDLCO), were not significantly different in the three groups at the time of diagnosis of fibrosis and cancer (data not shown).

**Fig. 1:**
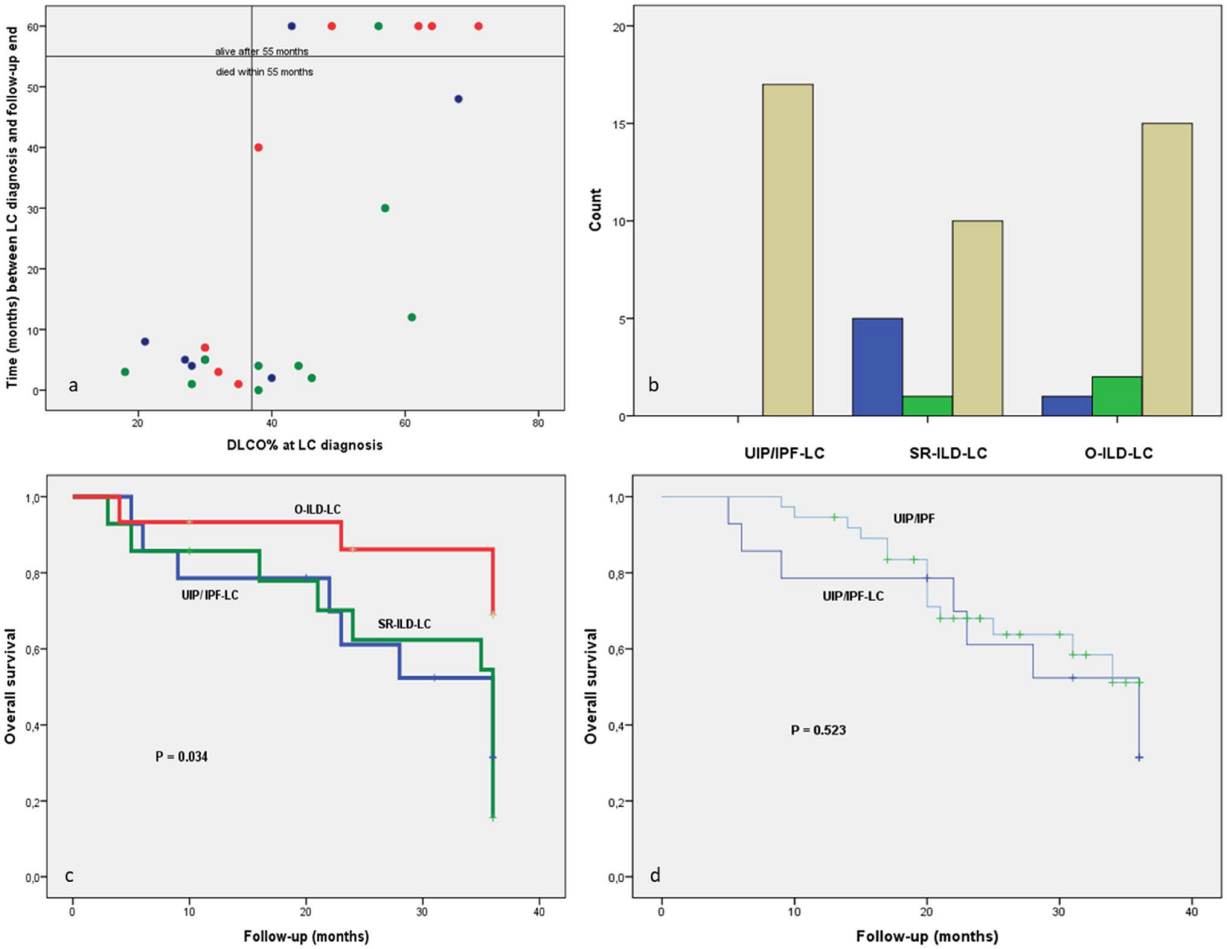
**a** Correlation between interval time ‘LC diagnosis and follow up end’ and DLCO% in the three groups: UIP/IPF-LC (blue circles), SR-ILD-LC (green circles), O-ILD-LC (red circles). **b** Localization of lung cancer in the three groups: UIP/IPF-LC, SR-ILD-LC, O-ILD-LC. Blue columns: central cancer not in fibrotic area; green columns: peripheral cancer not in fibrotic area; beige columns: peripheral cancer in fibrotic area. **c** Survival in the three groups of patients: UIP/IPF-LC, SR-ILD-LC, O-ILD-LC. **d** Survival in patients with UIP/IPF with and without lung cancer (LC)

A remarkable correlation was found between the time interval ‘LC diagnosis and end of follow-up’ and DLCO% (*p* = 0.006): patients with DLCO% < 38% showed survival < 10 months, irrespective of group. All patients with survival > 55 months showed DLCO% > 40% (shown in Fig. 1a).

### Lung cancer histology

Adenocarcinoma was the most frequent histological subtype in all three groups, with 13 cases in the UIP/IPF, nine in the SR-ILD and 13 in the O-ILD group Table 2.

**Table 2:** Histologic findings of lung cancer

Parameter	UIP/IPF-LC (number)	SR-ILD-LC (number)	O-ILD-LC (number)	p UIP/IPF-LC vs SR-ILD-LC
				p UIP/IPF-LC vs O-ILD-LC
				p SR-ILD-LC vs O-ILD-LC
*Istotype*				
Squamous carcinoma	3	2	2	0.105
Adenocarcinoma	13	9	13	0.338
Other	1	6	4	0.601
*Grade*				(G1 + G2 vs G3 + G4)
G1	1	1	1	
G2	1	4	7	0.210
G3	7	3	4	0.090
G4	1	3	3	0.950
*Localization*				
Central	0	5	1	0.022
Peripheral	17	12	17	0.514
				0.061
*Localization*				0.007
In advanced fibrosis	17	10	16	0.257
Unrelated with fibrosis	0	6	2	0.070

Abbreviation *UIP/IPF-LC* pattern usual interstitial pneumonia with lung cancer, *SR-ILD-LC* smoking-related interstitial lung diseases with lung cancer, *O-ILD-LC* other interstitial lung diseases with lung cancer, *G* grade

Grade 1 and 2 differentiation was more frequent in the O-ILD-LC (8/15) than the UIP/IPF-LC group (2/10) but the difference was not significant (*p* = 0.09).

Lung cancer was peripheral in most patients (17/17 in UIP/IPF-LC, 12/17 in SR-ILD-LC, 17/18 in O-ILD-LC) with a significant difference between the UIP/IPF-LC and SR-ILD-LC groups (*p* = 0.022). It developed in areas of advanced fibrosis in all patients with UIP/IPF-LC, in 10/18 cases with SR-ILD-LC and in 16/18 cases with O-ILD-LC. The difference between UIP/IPF-LC and SR-ILD-LC was significant (*p* = 0.007) (shown in Fig. 1b).

Bio-molecular target expression was investigated in too few patients to detect significant differences.

### Survival

In the population with lung cancer, survival 36 months after diagnosis of fibrosis was significantly greater for O-ILD-LC than for UIP/IPF-LC and SR-ILD-LC patients (*p* = 0.034), with no significant difference between the last two groups. Survival at 36 months was 70% for O-ILD-LC, 30% for UIP/IPF-LC and 10% for SR-ILD-LC patients (shown in Fig. 1c).

When we compared overall survival in fibrosis groups with and without lung cancer (UIP/IPF-LC vs UIP/IPF, SR-ILD-LC vs SR-ILD, O-ILD-LC vs O-ILD), there were no remarkable differences except for the SR-ILD groups, where carcinoma significantly affected prognosis (*p* = 0.016). In particular, comparing UIP/IPF groups with and without cancer, we observed that mortality was higher in the first 10 months after diagnosis of lung cancer, whereas it was similar in both groups at 30 months (shown in Fig. 1d).

## Discussion

In this study, we compared the clinical and histological characteristics of 53 patients with lung cancer and pulmonary fibrosis divided into three groups: UIP/IPF-LC (17 cases), SR-ILD-LC (17 cases) and O-ILD-LC (19 cases). Survival in these three groups was compared with survival in three control groups that did not have a history of lung cancer: UIP/IPF (38 cases), smoking-related ILD (6 cases), other ILD (21 cases). Age at diagnosis of lung fibrosis and lung cancer was higher in the UIP/IPF-LC group and lower in the O-ILD-LC group. IPF patients typically had an average age of 60 years and only those with familial IPF were younger, whereas age at diagnosis varied in patients with other ILDs [16].

A clear prevalence of male smokers or ex-smokers was observed in all groups. This finding is evidence that smoking plays a role in oncogenesis, irrespective of fibrosis type. In fact, lower immune surveillance and chronic inflammation with production of oxygen free radicals are reported to promote carcinogenesis in smokers [9].

The prevalence of males is in line with the literature on UIP/IPF and SR-ILD [16]. In the O-ILD group, the choice of including patients with pathologies having different incidences in males and females influenced the results. Some ILDs are more frequent in women (idiopathic NSIP, sarcoidosis) and others are more common in men (HP, environmental exposure).

Lung function did not differ significantly in any group but there was a close link with survival: DLCO% < 38% was predictive of poor outcome (shown in Fig. 1a). DLCO% has been reported to be predictive of prognosis in UIP/IPF and other progressive fibrosing ILDs [17, 18]. This parameter indicates the capacity, impaired by fibrosis, for gas exchange at the alveolar–capillary membrane [19].

Adenocarcinoma was the prevalent type of lung cancer in all three groups, whereas squamous and undifferentiated carcinomas were rare. This is in contrast with literature on lung cancer that indicates a prevalence of squamous cell carcinoma in patients with UIP/IPF-LC. This finding could be linked to a limited number of case studies or to genetic or exposure differences in the Italian population. We know that squamous cell carcinoma was prevalent in the past, but in recent years, pathologists have observed an increase in adenocarcinoma as a lung cancer type, probably related to the different smoking options and the availability of modern filtered cigarettes [20].

**Fig. 2:**
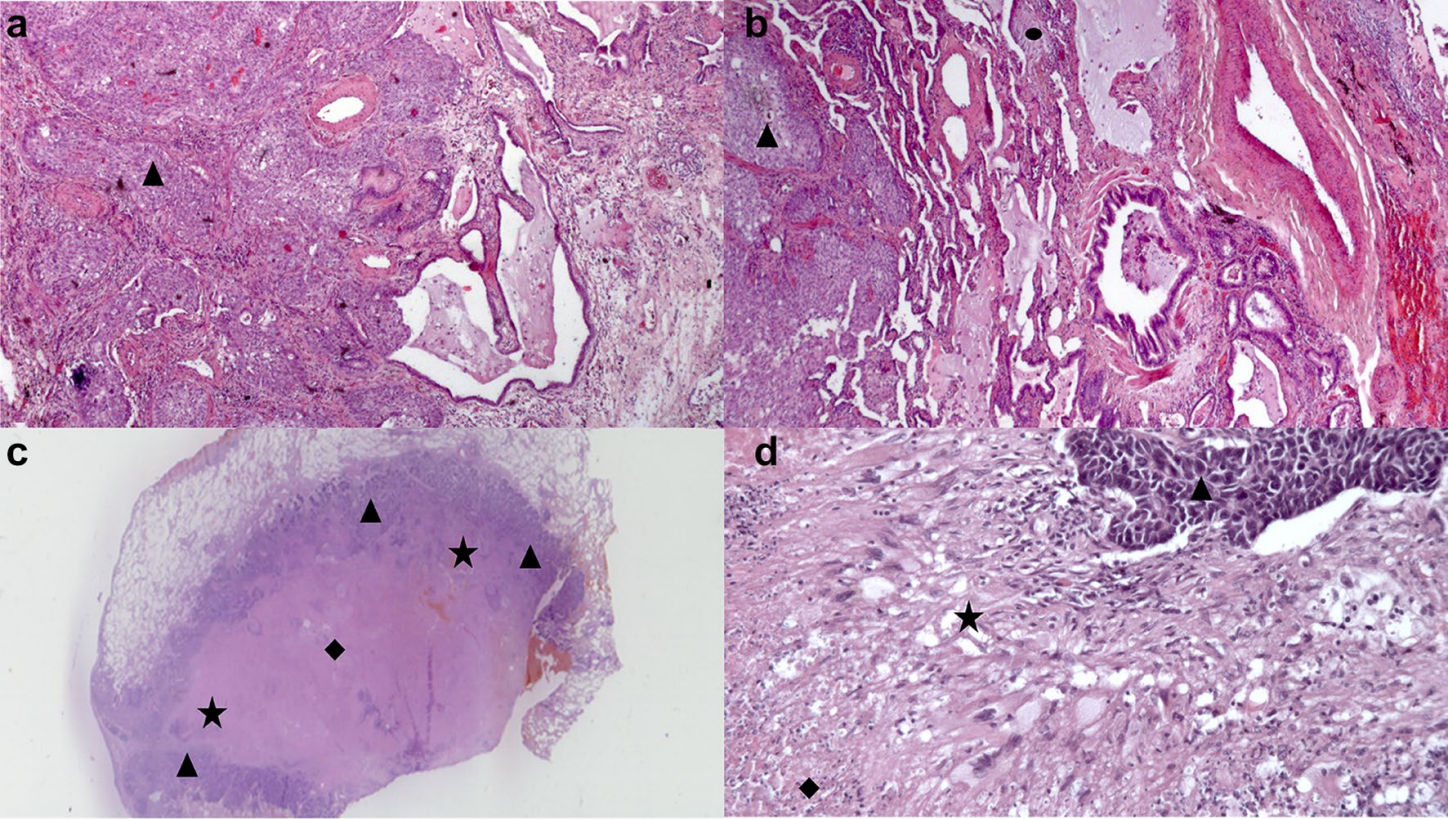
**a**, **b** UIP/IPF-LC, Fibroblastic Focus (*filled circle*). lung cancer (*filled triangle*) in honeycombing area. **c**, **d** O-ILD-LC, rheumatoid nodule. Lung cancer (*filled triangle*) at periphery of the nodule, histiocytic and lymphoid cells (*filled star*), fibrinoid necrosis (*filled diamond*)

In patients with UIP/IPF-LC, the cancer develops in the lung periphery and in an advanced fibrosis context, i.e., in areas with honeycombing or transitional fibroblastic foci [2] (shown in Figs. 1b, 2a and 2b). This result highlights that the fibrotic and oncogenic mechanisms are strictly connected in IPF because the neoplasm grows in fibrotic areas, whereas it doesn’t always happen in SR-ILD.

This is probably related to release of oncogenic factors, like transforming growth factor beta (TGF beta), involved in transformation of fibroblasts into myofibroblasts, in transition areas, to genetic mutations like p53 mutation [21] and to abnormal expression of mRNAs [22]. Researchers have also described abnormal activation of the Wnt/beta-catenin pathway in lung cancer and IPF, with consequent resistance to apoptosis [23].

These findings were also observed in the O-ILD-LC group, where cancer developed in a context of fibrosis and was largely peripheral, in line with the hypothesis that inflammatory cytokines (TGF beta) act as oncogenic factors that promote the development of cancer [9].

Figure 2c and d shows this strong relationship [24]. In the case of rheumatoid arthritis, neoplastic cells form a rim around lymphocytes and histiocytic inflammatory infiltrate surrounding a rheumatoid nodule.

In contrast, in one third of SR-ILD-LC cases, the neoplasm was central and was not linked to fibrotic injury. This could point out a different oncogenic mechanism in these patients. In fact, in this group, the oncogenic process could be independent of the fibrotic process, because it arises from airway epithelial cells In fact, cigarette smoke damages small as well as central airways, where epithelial remodeling and sub-epithelial fibrosis with infiltration of the mucosa and submucosa by inflammatory cells can be observed [25].

Survival time was longer in the O-ILD-LC group than in the UIP/IPF-LC and SR-ILD-LC groups. The better prognosis of O-ILD-LC patients was probably due both to a good response of the autoimmune/inflammatory disease to therapy, and low-grade lung cancer; however, the group was not homogenous, so this could influence the result.

Survival at 36 months was similar in O-ILD groups with and without lung cancer. This could indicate that lung cancer did not influence survival, although the follow-up was too short to document any deterioration in the prognosis of O-ILD cases with cancer.

Likewise, no differences in survival were found between IPF patients with and without LC. This is presumably related to the advanced stage of fibrotic disease in the patients enrolled; in fact, IPF was itself an independent negative prognostic factor for death [11] (shown in Fig. 1d).

The only group in which we documented a worse impact of LC on prognosis was the SR-ILD-LC group. However, the same group without LC consisted of only six cases, and although the difference was significant, further confirmation is necessary.

## Conclusion

Interstitial lung disease is a risk factor for lung cancer. Various pathogenetic pathways are responsible for carcinogenesis and certain factors, such as chronic inflammation, epithelial–mesenchymal transition, smoking and genetic alterations, can be linked to its onset.

We reported clinical and histological characteristics and the survival of 118 patients with pulmonary fibrosis (IPF/UIP, SR-ILD, O-ILD), with and without lung cancer. Our results confirm that the oncogenic mechanism is closely linked to fibrotic and inflammatory processes in IPF, but not in SR-ILD, in which different pathogenetic mechanisms could be involved. Moreover, adenocarcinoma was the most frequent histotype. In contrast with the literature, we found that the development of carcinoma in UIP/IPF does not necessarily affect survival, unlike in SR-ILD, showing the lack of prognostic impact of lung cancer in these patients and confirming that IPF has a prognosis that is worse than that of most cancers. This is an interesting finding, worthy of further investigation.

## Data Availability

The records extracted for use in this study will be fully anonymized. The data presented in this study are available on request to the corresponding author.

## Abbreviations

LC: Lung cancer
ILD: Interstitial lung diseases
UIP: Usual interstitial pneumonia
IPF: Idiopathic interstitial pneumonia
UIP/IPF: Usual interstitial pneumonia/idiopathic interstitial pneumonia
UIP/IPF-LC: Usual interstitial pneumonia/idiopathic interstitial pneumonia with lung cancer
ILD-LC: Interstitial lung diseases with lung cancer
SR-ILD: Smoking-related interstitial lung diseases
SR-ILD-LC: Smoking-related ILD with lung cancer
O-ILD: Other interstitial lung disease
O-ILD-LC: Other ILD with lung cancer
FVC: Forced vital capacity
DLCO: Diffusing capacity of the lung for carbon monoxide
HRCT: High-resolution computer tomography
TGF: Beta transforming growth factor beta
